# Readiness for Firefighting: A Heart Transplant Patient’s Quest to Return to Work

**DOI:** 10.3390/jcm8030378

**Published:** 2019-03-18

**Authors:** Darren E. R. Warburton, Arlana Taylor, Veronica K. Jamnik, Norman Gledhill, Shannon S. D. Bredin

**Affiliations:** 1 Cardiovascular Physiology and Rehabilitation Laboratory, University of British Columbia, Vancouver, BC V6T 1Z4, Canada; arlana@shaw.ca; 2 Physical Activity and Chronic Disease Prevention Unit, University of British Columbia, Vancouver, BC V6T 1Z4, Canada; shannon.bredin@ubc.ca; 3 Kinesiology and Health Science, York University, Toronto, ON M3J 1P3, Canada; ronij@yorku.ca (V.K.J.); ngledhil@yorku.ca (N.G.); 4 Laboratory for Knowledge Mobilization, University of British Columbia, Vancouver, BC V6T 1Z4, Canada

**Keywords:** transplantation, cardiac rehabilitation, return to work, physically demanding occupations, bona fide fitness requirements

## Abstract

Heart transplantation patients generally demonstrate exercise capacities that are below the minimal standards for firefighting. Therefore, it is unlikely that heart transplantation patients will receive medical and/or employer clearance for active duty. We report a case of a firefighter who sought to return to full-time active duty following heart transplantation. We examined his physiological readiness to return to work during occupation-specific testing. Remarkably, the patient was able to meet the minimal requirements for full active firefighting. This finding provides direct evidence to support the potential of transplant patients returning to active duty in physiologically demanding occupations.

## 1. Case Presentation

Cardiac transplant patients are often faced with a series of unique challenges that reduce their capacity to exercise [1]. Several factors have been associated with this exercise intolerance including prolonged inotropic support before transplantation, long-term deconditioning, chronic cardiac denervation, and the effects of immunosuppressive therapy [1]. Research has indicated that heart transplant patients are limited during exercise conditions by impaired preload, heart rate, and vascular reserves [2]. Peripheral maladaptations (as a result of prolonged deconditioning and immunosuppressive therapy) also appear to play key roles in the decreased aerobic exercise potential and/or musculoskeletal fitness of cardiac transplant patients [3,4,5].

The ability to return to work is a key factor in the transplant patient’s overall satisfaction and quality of life [6]. Unfortunately, return to work rates are exceptionally low for those who work in physically demanding occupations [6]. Heart transplant patients appear to have difficulty receiving medical and employer clearance for return to work, particularly in safety-related occupations (such as firefighting and police work) where safe and efficient job performance is critical to life and property under emergency circumstances.

The physiological requirements and inherent risks associated with safety-related occupations have created unique challenges for the employee and employer alike. Both the 1999 Meiorin Decision and Bill C-45-Section 217.1 of the Criminal Code of Canada (2004) impose significant legal obligations upon organizations to ensure the safety of their personnel and the general public. The Criminal Code of Canada (2004) states that “everyone who undertakes, or has authority to direct how another person does work or performs a task is under legal duty to take reasonable steps to prevent bodily harm to that person, or any other person, arising from that work or task.” As such, emergency-related physically demanding occupations have routinely established mandatory fitness criteria for applicants [7]. Moreover, annual evaluations of incumbent firefighters and police officers are often compulsory [8]. The basic tenant behind these requirements is that individuals who cannot meet the minimal requirements for critical job-related emergency tasks pose an increased risk to themselves, co-workers, and the general public alike [7,8].

Heart transplantation patients often exhibit maximal aerobic power (VO_2_ max) values (i.e., 20–25 mL/kg/min [1]) that are well below the minimal requirements for firefighting (i.e., 42–45 mL/kg/min (approximately 12 METs) [7,8]) creating a major barrier for return to front-line active duty. Moreover, transplant patients may have difficulty receiving medical clearance for return to work job-related fitness testing owing to concerns regarding their ability to complete strenuous occupation-related tasks. Consequently, a large majority of heart transplantation patients from physically demanding occupations fail to return to work [6]. Therefore, the primary purpose of this case study was to examine the ability of a heart transplant patient to meet the minimal requirements for firefighting duty in order to return to active duty.

## 2. Methodology

We evaluated a 44-year-old male structural firefighter who underwent heart transplantation and sought to return to work one year later. In August 2006, the otherwise healthy client developed shortness of breath, a persistent dry cough, fatigue, and other cold-like symptoms. The client reported a reduced capacity for carrying out activities of daily living (i.e., reduced functional status). Further evaluation (including echocardiography) revealed idiopathic dilated cardiomyopathy and end-stage heart failure (viral etiology). In November 2006, the patient left active firefighting duty as the result of his medical condition. He was added to the donor transplant list in January 2008 and received a heart transplant approximately 2 weeks thereafter. The duration from time of diagnosis to transplantation was approximately 1.5 years.

During the one-year post-transplantation follow-up (February 2009), the patient exhibited moderate pericardial effusion; however, the level of pericardial effusion was significantly lower than that observed immediately post-transplantation. In February 2009, he was on the immunosuppressants, tacrolimus and mycophenolate mofetil in addition to a series of other medications (Table 1). Baseline blood tests, resting heart rate and blood pressure values were generally within normal limits (Table 1). The patient underwent a dobutamine stress echocardiography test with incremental dosages of 50 to 80 mcg/kg/min. His resting heart rate was 85 bpm and his resting blood pressure was 116/79 mmHg prior to the test increasing to 141 bpm (81% of age predicted maximum heart rate) and 152/75 mmHg at the highest dosage of dobutamine. There were no abnormalities in wall motion, ejection fraction, and left ventricular volumes. The patient also underwent coronary angiography, which revealed normal coronary vessels.

The patient completed written informed consent and ethical approval (H05-50260) was obtained through the Clinical Research Ethics Board at the University of British Columbia. The patient has provided written consent to present the findings in this report. Medical clearance to participate in the fitness testing battery was provided by the patient’s treating physician.

Unique to this case, in order to return to work the participant was required to demonstrate the ability to complete a job-related test (i.e., the Fitness York Incumbent Fire Fighter Assessment Test) developed in accord with the Supreme Court of Canada Meiron Decision to qualify as a bona fide occupational requirement in addition to receiving medical clearance. This assessment measures the ability to sustain the repeated performance of critical physically demanding firefighting tasks, and meets the legal requirements put forward by the Criminal Code of Canada requiring employers to ensure the safety of their personnel, and the general public.

The incumbent protocol takes into account the past experience of incumbent firefighters while maintaining the due diligence responsibility of management (employers) concerning the safety of the firefighter, co-workers, and the public. The Fitness York Incumbent Fire Fighter Assessment Test provides an objective assessment of the occupation-specific fitness of incumbent firefighters or incumbent firefighters who are returning to work following a period of modified duty as the result of injury or disease [9]. This is a modification of the Fitness York Applicant Fire Fighter Assessment [7,8]. The incumbent battery requires firefighters to complete the same job-related firefighting tasks included in the applicant protocol; however, these tasks are completed in a continuous timed circuit. We chose this battery of testing owing to its reliability, widespread usage, supporting of scientific literature, and ability to discriminate those applicants and incumbent firefighters that are able to meet the physical demands of front-line firefighting. The Fitness York Incumbent Fire Fighter Assessment Test involves the following simulated firefighting tasks completed in a circuit with the client wearing a weighted vest (32 lb) and ankle weights (4 lb on each ankle) including:

1. Stair Climb: Wearing a weighted vest and ankle weights the client was required to climb up and down stairs (twice) that were 26 ft (7.9 m) in height.
2. Hose Carry/Stair Climb: After completing the stair climb, the participant was required to lift to an over-the-shoulder position then carry an 85 lb (38.6 kg) bundle of tied hose while climbing up and down stairs (once) that are 26 ft (7.9 m) in height.
3. Rope Pull: Using a rope, the participate was required to hoist and lower the weight (50 lb; 22.7 kg) of a 50 ft section of hose and nozzle a height of 65 ft (19.8 m).
4. Hose Advance/Drag: Via a strap positioned over the shoulder, the participant pulled a weighted sled (i.e., the weight of two sections of charged hose) a distance of 25 ft (7.6 m) in one direction then 25 ft (7.6 m) in the opposite direction back to the starting line.
5. Ladder Lift: The participant was then required to remove a 24 ft (7.3 m) extension ladder from wall-mounted brackets, lowering it to the floor, and then returning it to the original position.
6. Victim Drag: The participant was required to drag a 200 lb dummy by a handle located behind the neck a distance of 50 ft (15.2 m), weaving in and out of traffic cones placed every 8 ft (2.4 m).
7. Forced Entry: Using a 10 lb (4.5 kg) sledgehammer, the participant was required to repeatedly hit and move a heavily weighted tire a distance of 12 in (30.5 cm). This task simulates a forced entry through a door or wall.

Unique to this case study, we were also able to compare the participant’s findings to norm-referenced criterion test scores for four timed tasks (including hose carry/stair climb, rope pull, hose advance/drag, and victim drag) for applicant firefighters. The four timed job-related performance tests are each scored out of three (3) for a maximal total score out of 12 based on the mean and SD times for each task.

The participant completed a symptom limited, incremental to maximal exercise treadmill test with metabolic monitoring (K4b2 Cosmed, Roma, Italy) to determine VO_2_ max. A supramaximal confirmatory stage was used to support the attainment of VO_2_ max. Often incumbent firefighting tests are terminated when firefighters meet the minimum aerobic fitness requirement. However, in this case study, once the minimal level of aerobic fitness was met, we asked the participant to continue until VO_2_ max was achieved. This slight modification was done to meet both the requirements of the testing battery and the need of the medical team to have an objective indicator of VO_2_ max.

Prior to the completion of the exercise testing battery, the patient was familiarized with the testing procedures and engaged in a regimented exercise program consistent with preparing for active firefighting and physically demanding occupational testing batteries. This included moderate to vigorous intensity exercise training involving whole body muscular strengthening activities and aerobic activities. This training was conducted under the consultation of qualified exercise professionals (including a physiotherapist and exercise physiologist). The client exercised 3–5 days per week for 3 months prior to testing.

Unique to this case study, the participant was able to provide our team information from occupation-specific testing completed in 2002 and 2003 to allow for an objective comparison of his pre- and post-transplant occupation-specific findings. In 2002, the participant completed the Department of National Defence occupation-specific circuit involving 10 firefighting tasks [10,11]. At this time, the client also completed the University of Alberta Firefighter applicant test consisting of the assessment of aerobic fitness and six job-related tasks (charged hose advance, rope pull, forcible entry, victim drag, ladder climb, and vehicle extrication) [12]. In 2003, the participant completed a firefighter specific health-related physical fitness assessment including estimated aerobic power (via the 1.5-mile run) and musculoskeletal fitness.

## 3. Results

The patient completed the bona fide occupational fitness testing in March of 2009. The participant successfully completed all of the simulated firefighting tasks with a time (i.e., 4:56 min) that was substantially faster than the minimal standard for active duty (i.e., 8:30 min). In this study, we were also able to compare his findings to applicant firefighters completing these job-specific tasks. In each event, the participant received a 3 out of 3 score. Thus, he received the highest possible score (i.e., 12 out of 12) and an “Excellent” rating for the firefighting specific tasks in comparison to standardized criteria based on applicant firefighters.

The patient demonstrated a VO_2_ max of 43 mL/kg/min (during an incremental treadmill test with metabolic monitoring), which is above the minimal bona fide fitness cutoff for firefighters. Thus, the patient’s aerobic fitness met the requirement for front-line duty and was consistent with age-predicted values (approximately 100% of predicted).

Based on these combined results, the patient received medical and employer clearance for return to full front-line active duty shortly after (i.e., within two months) completing the occupation-specific bona fide fitness testing battery. More than 10 years later, the patient remains an active firefighter.

The participant’s post-transplantation findings compared well to the occupation-specific testing that he had completed approximately 6–7 years previously. For instance, in 2002 the candidate received an excellent score on the Department of National Defence Circuit with an estimated VO_2_ max of 52.7 mL/kg/min. At that time, he also received a score of 21 out of 25 on the job-related University of Alberta Firefighter applicant fitness test. In 2003, he received “Excellent” ratings for musculoskeletal fitness measures (including muscular strength, muscular endurance, and flexibility) with a predicted VO_2_ max of 45.6 mL/kg/min.

## 4. Discussion

Transplant patients have varied occupational backgrounds including physically demanding occupations [6]. Despite consistently low return to work rates of heart transplant patients, the ability to return to work is well-established as an important factor in the transplant patient’s overall satisfaction and quality of life [6]. Transplant patients that are able to return to work have less depression and fewer illnesses than non-workers [13].

Many transplant patients seek to return to a normal lifestyle shortly after transplantation. Therefore, it is understandable that individuals from physically demanding occupations would seek to return to work duty after transplantation. However, current evidence indicates that patients who work after heart transplantation generally come from occupations that require less physical exertion [6]. There is limited evidence that specifically examines the return to work rates in people that engage in public protection and safety jobs. Police officers and firefighters are often included in blue-collar occupations, despite more classically being defined as “grey collar” workers. Approximately 61% of heart transplant patients who do not work following transplantation come from more physically demanding blue-collar occupations [6].

It remains to be determined why physically demanding jobs are associated with lower return to work rates. There does appear to be hesitation amongst employers and transplantation specialists to allow transplantation patients (particularly heart transplant patients) to return to physically demanding occupations. It is hoped that this particular case will address directly and clarify this inequity.

Patients who have undergone orthotopic heart transplantation generally exhibit exercise capacities that are 40–60% of predicted [1,14]. Although VO_2_ max is improved from pre-transplant values (approximating 12–15 mL/kg/min) [15,16], it is not uncommon for heart transplant patients to have peak or maximal values of 20–25 mL/kg/min [1]. Therefore, the aerobic capacities of cardiac transplant patients are generally well below the established standard (bona fide) requirements for firefighting and other physically demanding occupations such as police work (which approximate from 42 to 45 mL/kg/min) [7,8]. Thus, based on their aerobic fitness many (if not most) transplant patients would not be considered eligible by their employers for active duty. Moreover, transplant patients may have difficulty receiving medical clearance for return to active duty owing to a lack of information regarding their ability to complete occupation-specific tasks that are often of a strenuous nature. This may explain (in part) why a large majority of heart transplantation patients from physically demanding occupations fail to return to work.

Unique to this case, in order to return to work the participant was required to demonstrate the ability to complete occupation-specific tasks in addition to receiving medical clearance for strenuous physical activity. It is important to highlight that medical clearance was also related (in part) to the patient’s ability to complete a stress test (achieving a sufficient VO_2_ max), and the various occupation-specific activities required of incumbent firefighters. To ensure that the client met the international and Canadian standards for firefighting and also to reduce the potential legal liability associated with this patient returning to work, we (in collaboration with the employers and medical team) chose to employ the bona fide fitness requirements established by Fitness York. This fitness battery (consisting of testing for both incumbent and applicant firefighters) is a bona fide occupational requirement for firefighting with overwhelming empirical data supporting its ability to meet the burden placed upon employers by the Criminal Code of Canada to ensure the safety of their personnel, and the general public. Each year thousands of incumbent or applicant firefighters must complete this mandatory test battery to determine their ability to meet the physical demands of firefighting. The fact that the current patient was able to meet the minimal requirements for aerobic fitness and excel in passing the requirements for the occupation-specific activities is truly remarkable. Also, of importance was the finding that he was able to achieve similar aerobic fitness and job-related physical performance ratings (i.e., excellent) to that observed 6 or 7 years prior to transplantation.

This case study provides further evidence that heart transplant patients can achieve aerobic capacities that meet and exceed the minimal requirements for active duty in physically demanding public safety and protection occupations. This is consistent with other research demonstrating the capacity of heart transplant patients to enhance their aerobic fitness levels to predicted or even above predicted values. For instance, Haykowsky and colleagues recently documented highly trained heart transplant patients with aerobic capacities well above that predicted for the general population and their medical condition [17]. The ability to engage in routine exercise training is important for these patients achieving superior aerobic fitness; however, other factors such as length of time of symptomatic heart failure prior to transplantation and etiology of heart failure likely play a role. In the present case study, the relatively short duration, the cause of heart failure (viral myocardiopathy), and the ability to engage in regimented moderate to vigorous exercise training likely played key roles in his ability to attain fitness levels meeting the occupational fitness requirements. Importantly, this client remains an active firefighter more than 10 years after his transplantation demonstrating the ability of heart transplant patients to return and remain at work in physically demanding occupations. It is also important to highlight that consistent with current recommendations the client also takes special measures to reduce infection risk while active firefighting by limiting the exposure to infections (e.g., frequent hand washing, avoiding close contact with infected individuals), being aware of the symptoms associated with infections, and seeking treatment as soon as possible (as required) [18,19].

## 5. Conclusions

Physically demanding occupations (such as firefighting and police work) can place significant stressors on the health and safety of employees. Employers are now required (by law) to ensure the safety and well-being of their personnel and the general public. This means ensuring that their employees are physically prepared to participate in full active duty. Therefore, employers are increasingly relying on bona fide occupational requirements to determine the candidate’s suitability to perform active duty. For most transplant patients, these requirements are above their capacity, likely explaining (in part) why so many transplant patients from physically demanding occupations fail to return to work. Moreover, in the transplantation field there is often hesitation to allow transplant patients (particularly heart transplant patients) to return to work in physically challenging occupations. In fact, it is currently unlikely that heart transplantation patients will receive medical and/or employer clearance to take part in return to work fitness assessments.

This inspirational case report indicates clearly that a heart transplant patient can achieve and exceed the minimal requirements for active front-line firefighting. It also provides direct evidence to support the need of medical teams to consider the physiological ability of each transplant patient before making decisions regarding their ability to return to active duty in physically demanding occupations. It is hoped that this particular case report will change current perceptions by employers and transplantation specialists to facilitate the return to work of individuals who are able to demonstrate their ability to meet the physical demands of their occupation.

### Key Take Home Message

With appropriate and individualized exercise training, heart transplant patients can achieve aerobic fitness levels that allow them to meet the minimal requirements for active duty in physically demanding occupations. Employers in physically demanding occupations should consider the unique attributes of each applicant, such that heart transplantation is no longer an absolute contraindication for return to full active duty.

## Figures and Tables

**Table 1:** Patient characteristics one-year post-transplantation.

**Measure**	**Value**
Height (cm)	188
Weight (kg)	83.5
Body Mass Index (kg/m^2^)	23.6
Body Surface Area (m^2^)	2.10
Resting Heart Rate (bpm)	85
Resting Blood Pressure (mmHg)	116/79
Resting End-Diastolic Volume (mL)	129.8
Resting End-Systolic Volume (mL)	50.2
Resting Stroke Volume (mL)	79.6
Resting Cardiac Output (L/min)	6.8
Resting Global Ejection Fraction (mL)	61%
High Density Lipoprotein Cholesterol (mmol/L)	1.72
Low Density Lipoprotein Cholesterol (mmol/L)	2.31
Total Cholesterol (mmol/L)	4.02
Triglycerides (mmol/L)	0.84
Hemoglobin (g/L)	127
Hemoglobin A1C (%)	5
White Blood Cell Count (x 10^9^/L)	4.0
Urea (mmol/L)	12.2
Creatinine (μmol/L)	89
**Medications**	
Immunosuppression: Calcineurin Inhibitor (Tacroliumus) and Anti-proliferative Agent (Mycophenolate Mofetil)	
Aspirin (ECASA)	
Angiotensin II Receptor Blocker (Atacand)	
Statin (Pravachol)	
Calcium Carbonate	
Folic Acid	
Multivitamin	
